# The impact of technical failures during cultivation of an inclusion body process

**DOI:** 10.1007/s00449-019-02158-x

**Published:** 2019-07-02

**Authors:** Alexander Pekarsky, Vanessa Konopek, Oliver Spadiut

**Affiliations:** Institute of Chemical, Environmental and Bioscience Engineering, Research Area Biochemical Engineering, Technische Universität Wien, Gumpendorfer Strasse 1a, 1060 Vienna, Austria

**Keywords:** Inclusion body, Technical failure, *Escherichia coli*, Upstream process, Downstream process

## Abstract

In biotechnological processes, technical failures in the upstream process often lead to batch loss. It is of great interest to investigate the empirical impact of technical failures to understand and mitigate their impact accurately and reduce economic damage. We investigated the impact in the upstream and downstream of a recombinant antibody fragment inclusion body production process chain to provide integrated empirical data and knowledge. First, we provided a reproducible process chain that yielded high inclusion body content, high specific product titer, and a refolding yield of 30%. The inclusion body downstream proved to be of high reproducibility. Through the intended introduction of technical failures, we were not only able to shed more light on the empirical responses in the upstream and downstream, but also on process-boosting parameters that would have been neglected. Herein, a short increase in temperature during the cultivation clearly increased the refolding yield.

**Electronic supplementary material:**

The online version of this article (10.1007/s00449-019-02158-x) contains supplementary material, which is available to authorized users.

## Introduction

Process design and development for new recombinant proteins is often complex, especially when therapeutic use is targeted. The United States’ Food and Drug Administration (FDA) has recognized the requirement for stricter risk-based controls during drug manufacturing processes. Therefore, an important initiative, entitled “Pharmaceutical cGMPs for the 21st Century: A Risk-Based Approach”, was launched in 2002. Shortly after, the “Process Analytical Technology” (PAT) followed that comprises recommendations to improve process understanding and analysis [1]. The FDA emphasizes that it is important to ensure high and robust product quality by real-time measurements and online monitoring, which also underlines that quality should not only be tested, but it should be built in. Later, the International Council for Harmonisation of Technical Requirements for Pharmaceuticals for Human Use (I.C.H.) released three fundamental frameworks: “Q8 Pharmaceutical Development” [2]; “Q9 Quality Risk Management” [3]; “Q10 Pharmaceutical Quality System” [4]. This resulted in the introduction of the “Quality by Design” (QbD) approach, released by the FDA together with the EMA (European Medicines Agency) [5–7]. QbD is a proactive and systematic approach for product and process development that is important to understand interconnections between product and process and minimizes risks using multivariate methods [8]. It requires the identification of a design and control space, in which the influence of known variations in critical process parameters (CPPs) on critical quality attributes (CQAs) or key performance indicators (KPIs) is understood [7].

Biotechnological processes are performed with technological equipment and sophisticated software tools [9]. Although process control, automation, and simulation are widely applied for biotechnological processes, several risks cannot be anticipated or are often tolerated if their probability of occurrence is below a given threshold (e.g., based on Failure Mode and Effects Analysis). Hardware- or software-based errors and also human-based errors can affect each unit operation in a biotechnological production process chain, which can lead to major deviations in product quantity and quality and even up to process termination [10, 11]. Thus, data-driven approaches for technical failures and process fault detection as also decision matrices are often implemented in standard biopharmaceutical processes [10, 11]. Due to insufficient knowledge or risk-based precautions, technical failures and process faults can often lead to process termination and batch loss. We believe that it is of great interest to investigate the empirical impact of technical failures on bioprocesses to understand and mitigate their impact accurately. Furthermore, this knowledge can help to avoid economic damage through batch loss. Technical failures can arise from numerous malfunctions during the upstream processing (USP). Some examples, their origin, and their impact are shown in Table 1 for the production of inclusion bodies (IBs) in *Escherichia coli*. The given examples were brought together from experiences in our research group and expert knowledge. IBs are formed in the cytosol of *E. coli* and they usually consist of aggregated, insoluble target protein that is misfolded or partially unfolded, leading to no or reduced activity [12, 13]. Their formation is mostly dependent on the used promoter system and strength [14], the target protein class [12], and the process conditions [15, 16]. However, the misfolded/unfolded character of IBs makes formation kinetics, size distributions in the cytosol, and IB purity comparable between similar protein classes (e.g., [15, 17]) until the initial IB solubilisation procedure, in which protein specific conditions have to be considered.

**Table 1:** Technical failures in the upstream processing, their origin and impact on the bioprocess, the *E. coli* cell, and the inclusion bodies

Technical failures	Origin	Impact on bioprocess	Impact on cell	Impact on IB
**Interruption of aeration**	Inlet filter blocked Outlet filter blocked Gas mixer defect	No aeration Decrease in dO_2_ No offgas analysis Headspace pressure not controllable Decreased mixing (if stirrer is interconnected) Acidification of medium (organic acids)	Switch to anaerobic metabolism (e.g., [18, 19]) Formation of organic acids and ethanol (growth decrease) (e.g., [18, 20]) Cellular stress	Decreased product formation (e.g., [19])
**Interruption of feeding**	Feeding tube blocked Feeding pump defect Feed tank empty	No substrate Increase in dO_2_ Decrease in offgas CO_2_ Increase in offgas O_2_	Maintenance metabolism (e.g., [21]) No or reduced growth (e.g., [21])	Decreased product formation
**Overfeeding**	Feeding pump defect Feed concentration too high Change in feeding parameter	Accumulation of substrate and acetate Decrease in dO_2_ Increase in offgas CO_2_ Decrease in offgas O_2_ Increased base addition	Increased µ and q_S_ overflow metabolism (e.g., [22]) Increased O_2_ demand Cellular stress	Increased product formation (e.g., [16]) Decreased product formation (overflow metabolism) (e.g., [19])
**Failure in pH control**	Base tube blocked Base pump defect pH probe defect	Acidification of medium Cell dependent change in offgas signals	Change in metabolism (e.g., [23]) Decreased viability and growth (e.g., [23]) Cellular stress	Lower pH can improve IB titer and purity [15, 24] Low pH increases IB density (decreased solubility in DSP) (e.g., [25])
**Failure in temperature control**	Temperature probe defect Heat exchanger defect	Increase in temperature Decrease in dO_2_ Increase in offgas CO_2_ Decrease in offgas O_2_	Increased metabolic activity (if temperature increases) Increased probability of cell lysis or leakiness (e.g., [26]) Cellular stress	Impact on IB activity (e.g., [27, 28]) Impact on IB titer (e.g., [15, 28])
**Failure in agitation**	Stirring motor defect Aeration interrupted (if stirrer is interconnected)	Decreased mixing Decrease in dO_2_	Decreased substrate/O_2_ availability Medium heterogeneity → Stress (e.g., [18, 19, 29, 30])	Decreased product formation (e.g., [19])

It was assumed that the respective technical failures occur during the induction phase, in which target protein is produced and, therefore, represents the most critical process phase

In this study, we investigated the impact of common technical failures during the USP on (1) cell physiology of *E. coli* and (2) the downstream processing (DSP) and impurity of an antibody fragment, produced as intracellular IB product to provide an integrated understanding. Technical failures were manually introduced during the induction phase. We kept the resulting process deviation phase for approximately 1 h, as we expected that period to be required to detect and correct the failure. After that, we allowed the cells to regenerate under standard process conditions for at least another hour, before we analysed the effects. The produced IBs were analysed quantitatively in the DSP unit operations IB wash, IB solubilisation, and IB refolding. We found that technical failures, like presented in Table 1, do not necessarily require process termination and batch loss. Furthermore, our results provide indications that certain technical failures or parameter shifts can even have a positive impact on the IB DSP.

## Materials and methods

### Chemicals

All chemicals were purchased from Carl Roth GmbH (Vienna, Austria), if not stated otherwise.

### Strain and expression

The gene coding for a recombinant antibody fragment was cloned into the pET-28a(+) vector together with a kanamycin resistance and a stop codon downstream of the target sequence. Then, this target gene containing vector was transformed into an *E. coli* strain BL21(DE3). Cryo cultures were prepared in 25% glycerol and used for each fermentation.

### IB production process variance

Bioreactor cultivations are rather reproducible when performed under the same conditions, but IB processing, including all DSP unit operations, requires a high degree of human interaction. Therefore, we performed four parallel verification runs (C1–C4) to analyse the variance of the whole IB production process chain without the introduction of technical failures. The harvested biomass was disrupted and the IBs underwent the whole DSP. The variance of each parameter or unit operation was expressed as the average absolute error ($\varTheta$) with formula (1) and (2):

1 $${\text{average mean}} \left( {\bar{x}_{\text{ave}} } \right) = \frac{{\mathop \sum \nolimits_{i = 1}^{n} \bar{x}_{i} }}{n},$$

2 $$\varTheta = \left( {\frac{{\mathop \sum \nolimits_{i = 1}^{n} \left| {\bar{x}_{\text{ave}} - \bar{x}_{i} } \right|}}{n}} \right) \times \frac{{\bar{x}_{\text{ave}} }}{100} ,$$

*i* = respective cultivation of C1–C4 (*n* = 4; number of cultivations), and $\bar{x}_{i}$ = calculated average of respective parameter in cultivation *i*.

### Upstream process

Ten cultivations were performed (Table 2). For all cultivations, a preculture was performed. Each bioreactor cultivation was composed of a batch and a non-induced fed-batch phase to generate biomass followed by an induced fed-batch phase (induction phase) to produce the target protein as intracellular IBs.

**Table 2:** Performed cultivations with and without technical failures

Cultivation	Technical failure	Theoretical origin	Real origin	Total induction time
C1–C4	Reproducibility runs			8.4 h
C5	Reference run			8.1 h
C6	Failure in pH control	e.g., Base pump defect	pH control turned off	8.1 h
C7	Failure in *T* control	e.g., Heat exchanger defect	*T* control turned off	8.1 h
C8	Reference run			11.1 h
C9	Interruption of feeding	e.g., Empty feed tank	Feed pump stopped	11.1 h
C10	Overfeeding	e.g., Wrong feed concentration	Set higher *q*_s, Glc_ for feed addition control	11.1 h

The preculture medium was prepared according to [31]. Ingredients per litre were: 8.8 g d-(+)glucose monohydrate, 13.3 g KH_2_PO_4_, 4.0 g (NH_4_)_2_HPO_4_, 1.7 g citric acid, 1.2 g MgSO_4_·7 H_2_O, 0.1 g Fe(III)citrate, 0.0084 g EDTA, 0.0130 g Zn(CH_3_COO)_2_·2 H_2_O, 0.0045 g thiamine HCl, 0.1 g kanamycin sulphate, and 5 mL trace-element solution (TE). TE contained the following ingredients per litre: 0.0025 g CoCl_2_·6 H_2_O, 0.0150 g MnCl_2_·4 H_2_O, 0.0012 g CuCl_2_·2 H_2_O, 0.0030 g H_3_BO_3_, and 0.0025 g Na_2_MoO_4_·2 H_2_O. Each stock was sterilized separately by autoclavation or sterile filtration with a 0.2 µm filter. Batch media were similar to the preculture media, but contained 22 g L^−1^d-(+)glucose monohydrate and 0.1 g L^−1^ Antifoam PPG 2000 (Sigma-Aldrich, Austria). Feed media contained per litre: 400 g d-(+)glucose monohydrate, 18.18 g MgSO_4_·7 H_2_O, 0.03636 g Fe(III)citrate, 0.01182 g EDTA, 0.01455 g Zn(CH_3_COO)_2_·2 H_2_O, and 7.27 mL TE.

#### Preculture

Precultures were performed in shake flasks at pH 7.2 (set with 10 M NaOH) for 8–10 h at 37 °C and 230 rpm in an Infors HR Multitron shaker (Infors, Bottmingen, Switzerland). The liquid volume was set to 10–20% of the possible working volume of the shake flask to assure proper aeration.

#### Batch phase

Bioreactor cultivations were carried out in the Eppendorf DASGIP parallel 4 × 2.5 L working volume bioreactor system (Eppendorf AG, Hamburg, Germany) with a capacity of four vessels simultaneously. The system was equipped with a calibrated EasyFerm Plus pH probe (Hamilton, Reno, NV, USA) and a fluorescence dissolved oxygen electrode Visiferm DO425 (Hamilton, Reno, NV, USA) for dO_2_ online measurement. The cultures were aerated with 2.0 vvm dried air and offgas of the cultures was measured using an infrared cell for CO_2_ and a ZrO_2_ sensor for O_2_ concentration (Blue Sens Gas analytics, Herten, Germany). Batch media were inoculated with 10% of the final batch volumes from the precultures. Batch cultivations were performed at 35 °C, setpoint for pH was 7.2 (adjusted with 12.5% NH_4_OH), dO_2_ was set above 30%, and a constant agitation speed of 1200 rpm. If agitation was not sufficient to hold the dO_2_ above 30%, pure O_2_ was mixed with the dried air aeration. The end of the initial batch phase at 35 °C, and therefore, complete glucose consumption was indicated by an increase in dO_2_, a drop in offgas CO_2_, and an increase in offgas O_2_.

#### Non-induced and induced fed-batch phase

After the batch phase, the non-induced fed-batch phase started. Again, the temperature was held constant at 35 °C, the dO_2_ above 30% and the pH at 7.2. The feed was added at a specific substrate uptake rate (*q*_s, Glc_) of 0.3 g g^−1^ h^−1^ and a biomass yield (*Y*_X/S_) of 0.4 g g^−1^, based on prior optimization of the cultivation conditions to increase specific product titer (data not shown). The fed-batch phase ran until a dry cell weight (DCW) biomass concentration of approximately 35 g L^−1^ was reached. Prior to induction, the temperature setpoint was set to 30 °C for optimal induction. The temperature of 30 °C, the dO_2_ above 30%, and the pH at 7.2 were held constant during this phase, but not during all deviation phases due to technical failures. The cells were induced by adding a pulse of sterile IPTG to a final concentration of 1 mM, *q*_s, Glc_ was kept at 0.2 g g^−1^ h^−1^, and a *Y*_X/S_ of 0.35 g g^−1^ was used for feeding. The total induction time included the standard induction phase, the deviation phase, and the regeneration phase (Table 2).

#### Introduction of technical failure

Technical failures were introduced between 4 and 8.4 h of induction time (Table 2). To assure accurate results, we performed reference cultivations, C5 and C8, respectively, to each set of technical failure cultivations C6/C7 and C9/C10. The used DASGIP system made it possible to perform each set of cultivations in parallel. As can be seen from the total induction time in Table 2, C8–C10 were cultivated longer than the C1–C7. This longer induction time was based on the respective technical failures. The interruption of feeding and overfeeding was assumed to be classic technical failures that occur in the late or final stages of the induction phase. Especially, overfeeding is usually a problem with increasing induction times, because cellular performance is usually decreasing over time [32]. When no automated closed-loop feed addition is performed, substrate overfeeding is usually present, as the specific substrate uptake rate of *E. coli* decreases [32]. Therefore, the technical failures in C9 and C10 were introduced at the usual end of the induction time. Each deviation phase lasted approximately 1 h and was followed by at least 1 h of regeneration under standard induction conditions. The duration of the deviation phases were set to approx. 1 h to mimic the estimated time that is needed from detection to repair of the technical failure.

#### Sampling strategy

Samples were taken during the cultivations: at the beginning of the batch; start of non-induced fed-batch; start of induced fed-batch, start of deviation phase; end of deviation phase; during and at the end of the regeneration phase. IBs were harvested and processed at the end of each cultivation.

### Sample analysis

Dry cell weight (DCW) was determined by centrifugation of 5 mL culture broth (4000*g*, 4 °C, 10 min), washing the pellet once with 5 mL water, and subsequent drying for 72 h at 105 °C. Determination was performed in triplicates. OD_600_ of the culture broth was measured in duplicates using a spectrophotometer (Genesys 20; ThermoFisher Scientific, Vienna, Austria). Protein concentration of cell free supernatant was determined at 595 nm using the Bradford Protein Assay Kit (Bio-Rad Laboratories GmbH, Vienna, Austria) with bovine serum albumin (BSA) (protein standard; micro standard, liquid; P0914; Sigma-Aldrich, Vienna, Austria) as standard. Relative DNA content was measured as absorption at 260 nm with a NanoDrop-2000 (ThermoFisher Scientific, Vienna, Austria). Concentration of glucose and other metabolites was determined in cell free samples of the bioreactor cultivation by HPLC (Agilent Technologies, Santa Clara, United States) equipped with a Supelco guard column and a Supelco gel C-610H ion-exchange column (Sigma-Aldrich, Vienna, Austria) and a refractive index detector (Agilent Technologies, Santa Clara, United States). The mobile phase was 0.1% H_3_PO_4_ with a constant flow rate of 0.5 mL min^−1^ and the system was run isocratically. Calibration was done by measuring standard points in the range of 0.1–10 g L^−1^ glucose and metabolites (formate, ethanol, acetate). Along with the observed standard deviations for the measurements of DCW, glucose, and metabolites, the errors were propagated to the specific rates as well as to the yield coefficients.

### Downstream process

#### Homogenization of broth and isolation of inclusion bodies

The frozen cell pellet was resuspended in homogenization buffer (50 mM TRIS, pH 8.0) at a concentration of 10–20 g DCW L^−1^. Factor of wet cell weight (WCW) to DCW was previously determined as 3.89 ± 0.21 (*n* = 3). Homogenization was done at 1500 bar with 3 passages on a PandaPLUS 2000 (GEA Mechanical Equipment, Parma, Italy), which was shown to be sufficient for complete cell disruption [33]. Afterwards, the homogenized suspension was centrifuged at 15,650*g* for 20 min at 4 °C and the supernatant was discarded. The pellet was washed, centrifuged, and resuspended with deionized water at a concentration of 100 g WCW L^−1^ twice to remove host cell proteins from the IB pellet. To resuspend the pellet properly, a T-10 basic Ultra-Turrax (IKA, Staufen, Germany) was used at power level 5 for 30–60 s. After centrifugation, samples of the supernatant from the washing steps were taken for determination of total and target protein loss values. The washed IBs were stored at − 20 °C.

#### Solubilization of IBs

Solubilization of IBs was done with solubilisation buffer (0.05 M TRIS, 2 M Urea, pH 12) at a concentration of 100 g wet weight L^−1^ similar to examples from literature [34, 35]. The T-10 basic Ultra-Turrax was used at power level 5 for 30–60 s to suspend the IBs in the buffer. The solution was shaken at 80 rpm at room temperature for 1 h. The solution was centrifuged at 15,650*g* at 4 °C for 20 min to sediment insoluble artefacts. Prior to the refolding, a small amount of IBs from the respective cultivation was solubilised and analysed via size exclusion chromatography (SEC) HPLC to determine protein concentration, which was later used to determine the necessary solubilisate volume for the refolding. The solution, containing solubilised IBs, was called solubilisate. The final solubilisate with known protein concentration was used for refolding immediately.

#### Refolding

Refolding was done in sterile 50 mL tubes. The refolding buffer only contained 8% v/v glycerol to preserve protein stability and prevent protein aggregation [36]. No additives were further used in the refolding buffer, beside of residual urea and TRIS from the solubilisate addition. Prior optimization studies have shown that the very mild refolding buffer (deionized water and 8% v/v glycerol) was sufficiently working like TRIS containing buffers with various additives. Around 0.5 mL solubilisate and 1.0 mL solubilisation buffer were mixed and added to 38.5 mL precooled refolding buffer. Refolding was done in a total volume of 40 mL at 7 °C under light shaking at a protein concentration of around 0.5 g L^−1^. Samples were taken after 180 min and were snap-frozen in liquid nitrogen to prevent degradation and to preserve the refolding state until further analysis. Refolding samples were then stored at − 20 °C and analysed on HPLC within a week.

### Sample analysis

During the course of the study, we observed that protein concentration of the samples was more accurate and reproducible when analysed by SEC HPLC instead of Bradford assay. Due to a non-existing standard of the target protein, a BSA standard was used for the determination of protein concentration. Chromatographic analysis was done at 280 nm with the software Chromeleon 7 Chromatography Data System Version 7.2 SR5 (Thermo Scientific, Vienna, Austria).

#### Sample preparation

Samples of solubilisate and refolded target protein were prepared by the following: Snap-frozen samples were directly thawed for 3 min at 37 °C in a heating block and inverted carefully six times. Then, they were centrifuged for 2.5 min at 14,800 rpm in a micro centrifuge. 500 µL of the sample were transferred into an HPLC glass vial and measured immediately.

#### HPLC analysis of solubilisate samples

Solubilisate samples were measured with a BioBasic SEC-300 size exclusion column (Thermo Scientific, Vienna, Austria). The mobile phase was 4 M guanidine hydrochloride (Gdn-HCl), 0.05 M Bis–TRIS, 0.15 M NaCl, and pH 6.8. The injection volume was 10 µL and an isocratic flow of 0.1 mL min^−1^ was used for 30 min. All buffers were sterile filtrated and sonicated. Total areas and target peak areas were identified in the chromatograms to determine total and target protein concentration with a BSA standard calibration. BSA standards were prepared in solubilisation buffer to a concentration range of 1–100 g L^−1^.

#### HPLC analysis of refolding samples

Refolding samples were measured with an MAbPac™ SEC-1 size exclusion column 4 × 300 mm length (Thermo Scientific, Vienna, Austria). The mobile phase was 0.1 M sodium dihydrogen phosphate, 0.3 M NaCl, pH 6.8. The injection volume was 10 µL and an isocratic flow of 0.150 mL min^−1^ was used for 40 min. All buffers were sterile filtrated and sonicated. Total areas and target peak areas were identified in the chromatograms to determine total and target protein concentration with a BSA standard calibration. BSA standards were prepared in refolding buffer to a concentration range from 0.1 to 5.0 g L^−1^.

#### Determination of specific product titer

The product titer was not defined as the amount of IBs per biomass, but as the amount of soluble target protein in the solubilisate, which was derived from a defined amount of IBs and biomass. For the specific product titer determination, the DSP protocol was carried out until the solubilisation step. The specific amount of target protein was determined according to formula (3):

3 $${\text{Specific product titer}}\;\left[ {\frac{{{\text{mg target}}}}{{{\text{g WCW}}}}} \right] = \frac{{{\text{solu}}.\;{\text{buffer}}\;\left[ {\text{L}} \right] \times {\text{target peak}}\;\left[ {\frac{{\text{g}}}{{\text{L}}}} \right]}}{{{\text{weighed biomass in WCW prior to cell disruption}}\;\left[ {\text{g}} \right]}} \times 1000,$$

where solu. buffer = amount of solubilisation buffer used to solubilise IBs at a concentration of 100 g L^−1^.

#### Determination of refolding yield

Areas under the curve of each peak were used to calculate the protein concentration and refolding yield was calculated with formula (4):

4 $${\text{Refolding}}\;{\text{yield}} \, \left[ \% \right] = \frac{{{\text{target}}\;{\text{peak}}\;{\text{in}}\;{\text{refolding}} \left[ {\text{g}} \right]}}{{{\text{target}}\;{\text{peak}}\;{\text{in}}\;{\text{solubilisate}} \left[ {\text{g}} \right]}} \times 100.$$

## Results

### Process reproducibility

The impact technical failures in the USP can only be investigated properly, if the process is understood and under control. Therefore, we performed four cultivations (C1–C4) to test for process reproducibility in the USP and DSP. These cultivations represented the standard process for the production of our target protein as IB. Furthermore, the IBs of the final biomass underwent a classical IB DSP down to the final refolding step. Based on the results, we were able to understand the variance of each unit operation or phase, which was given by the absolute average error ($\varTheta$) (Table 3). The USP and DSP of our IB production process was generally reproducible in regards to the final biomasses and the physiological parameters (*µ*_max_, *q*_s, Glc_, *Y*_X/S_, $Y_{{{\text{CO}}_{ 2} /{\text{S}}}}$) in the USP (see Fig. S1 for exemplary process and physiology data of cultivation C1). The high $\varTheta$ for the *Y*_X/S_ in the induction phase was attributed to the human interaction during biomass determination. Furthermore, also the DSP yielded comparable results between the different IBs. However, we encountered relatively high standard deviations for each parameter in the DSP and, therefore, also for the specific product titer. Although IB processing was done carefully, we assumed that the deviations could be also attributed to the required human interaction in each laboratory scale DSP unit operation. Nevertheless, after the IB wash, we calculated that the final biomass consisted to 30% of IBs, which underlined that our induction parameters were chosen accurately to produce high amounts of intracellular IBs. The final refolding process resulted in comparable amounts of target protein.

**Table 3:** Results for the reproducibility runs in the upstream and downstream process

	C1	C2	C3	C4	$\varTheta$ [%]
**USP**					
*Batch phase*					
DCW_End_ [g L^−1^]	8.8 ± 0.9	9.8 ± 0.1	9.2 ± 0.1	9.8 ± 0.1	4.3
*µ*_max_ [h^−1^]	0.55	0.52	0.56	0.54	n.a.
*Y*_X/S_ [Cmol Cmol^−1^]	0.45 ± 0.20	0.47 ± 0.11	0.46 ± 0.03	0.50 ± 0.06	3.0
$Y_{{{\text{CO}}_{ 2} /{\text{S}}}}$ [Cmol Cmol^−1^]	0.36 ± 0.01	0.36 ± 0.00	0.37 ± 0.01	0.38 ± 0.01	8.2
C-balance [Cmol Cmol^−1^]	0.82 ± 0.21	0.86 ± 0.12	0.84 ± 0.05	0.89 ± 0.07	2.6
*Non-induced fed-batch phase*					
DCW_End_ [g L^−1^]	32.6 ± 0.2	35.5 ± 0.1	33.6 ± 0.4	33.6 ± 0.7	2.5
*q*_s, Glc_ [g g^−1^ h^−1^]	0.20 ± 0.00	0.22 ± 0.00	0.23 ± 0.00	0.22 ± 0.00	4.0
*Y*_X/S_ [Cmol Cmol^−1^]	0.53 ± 0.14	0.47 ± 0.16	0.45 ± 0.09	0.45 ± 0.15	5.6
$Y_{{{\text{CO}}_{ 2} /{\text{S}}}}$ [Cmol Cmol^−1^]	0.52 ± 0.01	0.44 ± 0.00	0.44 ± 0.00	0.42 ± 0.00	7.1
C-balance [Cmol Cmol^−1^]	1.05 ± 0.14	0.93 ± 0.17	0.89 ± 0.09	0.88 ± 0.15	6.0
*Induction phase*					
IPTG concentration [mM]	1.0	1.0	1.0	1.0	n.a.
Duration [h]	8.4	8.4	8.4	8.4	n.a.
DCW_End_ [g L^−1^]	42.5 ± 0.1	47.5 ± 0.9	44.8 ± 0.1	44.0 ± 0.8	3.2
*q*_s, Glc_ [g g^−1^ h^−1^]	0.21 ± 0.00	0.20 ± 0.00	0.22 ± 0.00	0.24 ± 0.00	4.6
*Y*_X/S_ [Cmol Cmol^−1^]	0.25 ± 0.11	0.40 ± 0.11	0.35 ± 0.03	0.24 ± 0.09	21.1
$Y_{{{\text{CO}}_{ 2} /{\text{S}}}}$ [Cmol Cmol^−1^]	0.52 ± 0.00	0.53 ± 0.01	0.51 ± 0.00	0.54 ± 0.00	1.9
C-balance [Cmol Cmol^−1^]	0.82 ± 0.11	1.03 ± 0.12	0.91 ± 0.04	0.82 ± 0.10	8.4
Specific product titer [mg g^−1^ WCW]	57 ± 9	67 ± 0	73 ± 15	75 ± 5	8.8
**DSP**					
*IB wash*					
Ratio target/total [–]	0.11 ± 0.02	0.10 ± 0.01	0.10 ± 0.02	0.08 ± 0.02	9.0
Ratio IB/BM [–]	0.28 ± 0.01	0.29 ± 0.01	0.29 ± 0.02	0.29 ± 0.04	1.5
*IB solubilisation*					
Duration [h]	1	1	1	1	n.a.
Ratio target/total [–]	0.49 ± 0.07	0.58 ± 0.05	0.51 ± 0.11	0.67 ± 0.07	11.1
*IB refolding*					
Duration [h]	3	3	3	3	n.a.
Refolding yield [%]	30 ± 6	31 ± 7	31 ± 6	33 ± 1	2.4
Ratio target/total [–]	0.21 ± 0.02	0.24 ± 0.03	0.23 ± 0.02	0.25 ± 0.04	5.4

Results are given for each phase in the upstream process. Batch phase for initial biomass accumulation, non-induced fed-batch (fed-batch) phase, and induced (induction) phase. Dry cell weight measurement errors were derived from triplicate measurements. Standard deviations from physiological parameters were derived from error propagation. Standard deviations from the specific product titer and the downstream process parameters were derived from duplicate processing and measuring. Shown downstream process focused on the inclusion body washing procedures (IB wash), the inclusion body solubilisation procedure (IB solubilisation), and the final refolding process (IB refolding). The absolute average error ($\varTheta$) was determined to gain insight on variation of the process parameters for each phase and unit operation

*n.a.* not applicable

### Introduction of technical failures in the induction phase

**Fig. 1:**
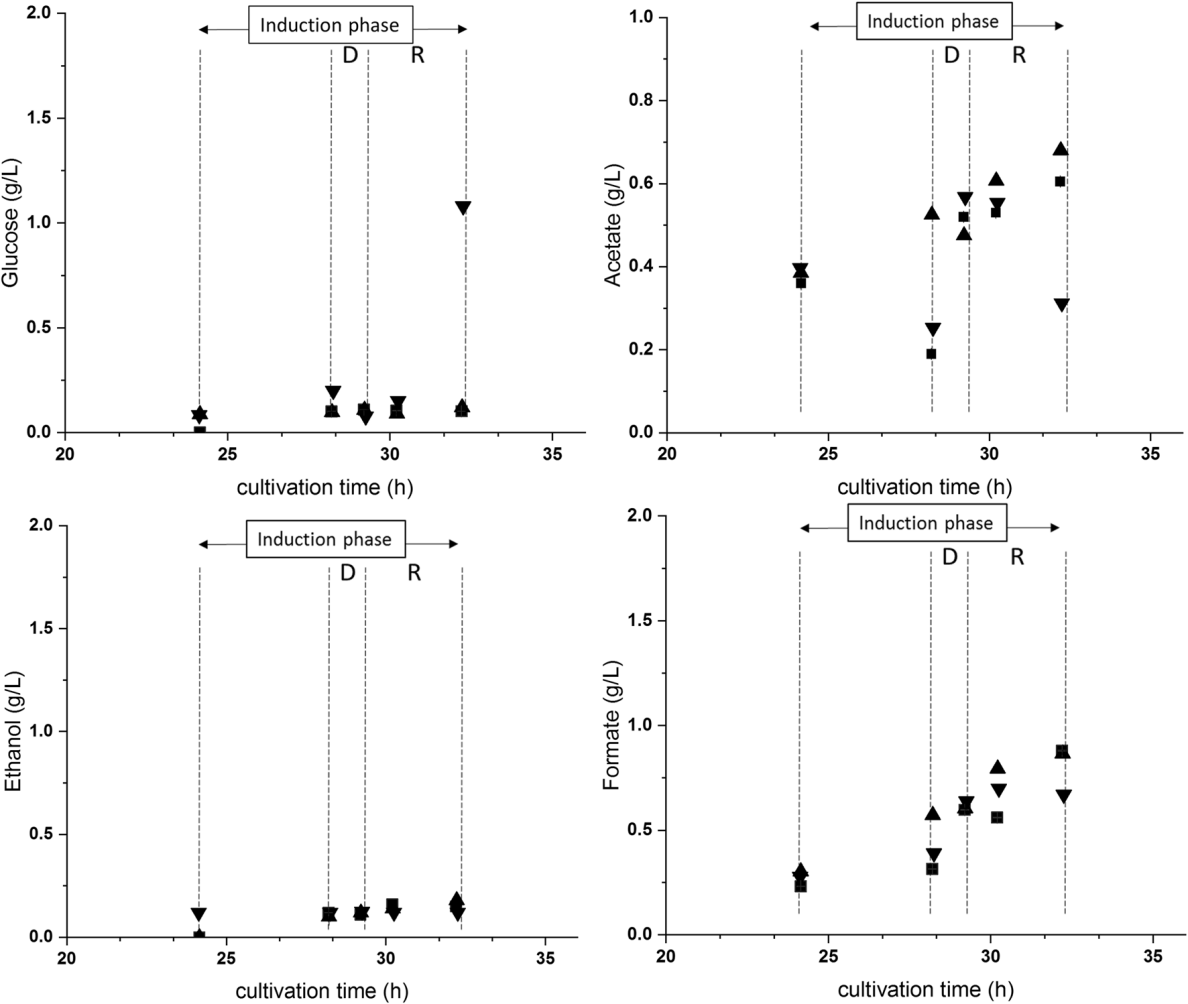
Monitoring of glucose and metabolite (ethanol, formate, and acetate) content in the cultivation broths of C5–C7. (Filled square) C5—reference run; (filled triangle) C6—failure in temperature control; (filled inverted triangle) C7—failure in pH control. Induction phase is shown. Deviation phase is shown as D and regeneration phase as R. The induction phase included the phases D and R

**Fig. 2:**
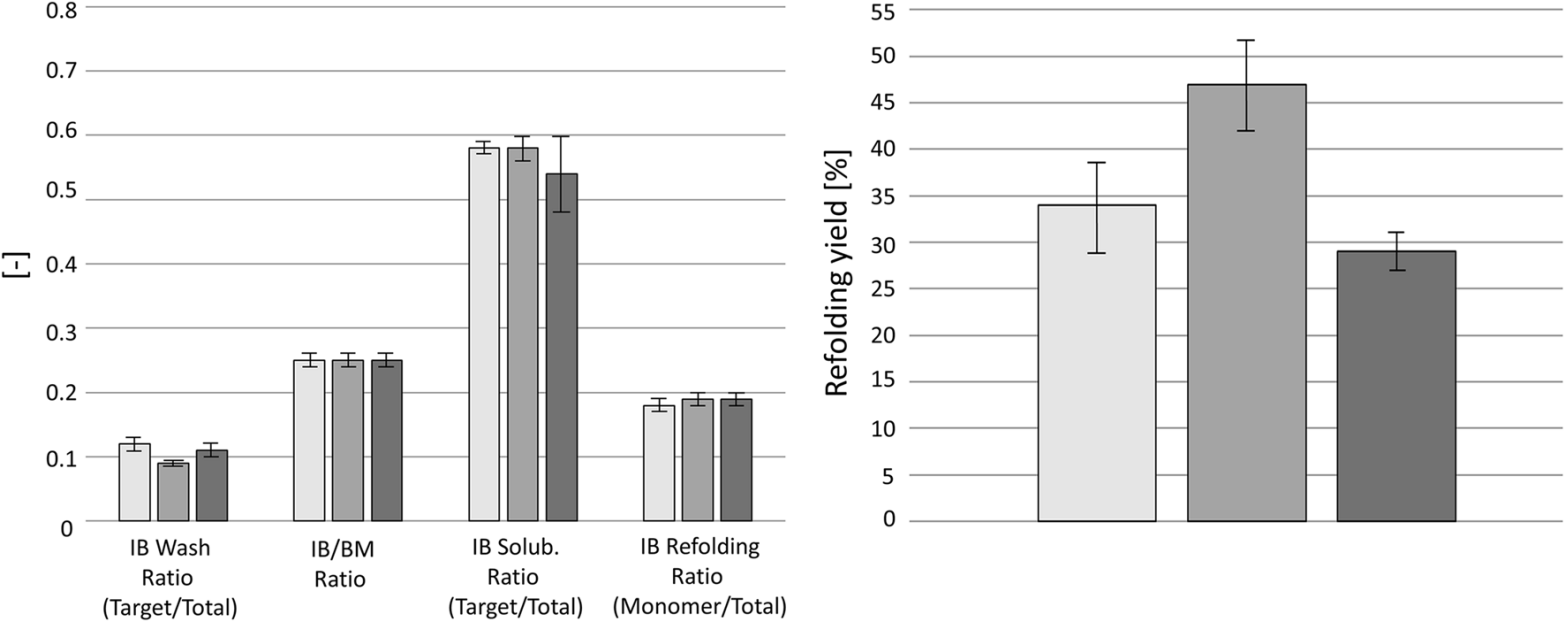
Evaluation of inclusion body downstream processing from cultivations C5—C7. Bars from left to right show results from C5—reference run, C6–failure in temperature control, and C7—failure in pH control. On the left, the results of the inclusion body wash procedure, the measured ratio of inclusion body to biomass after the inclusion body wash, the purity of inclusion body solubilisation, and the purity of the inclusion body refolding procedure are shown. On the right, the final refolding yield after 180 min of refolding is shown. Error bars were derived from duplicates for all shown results

In cultivation C6 and C7, the technical failures “failure in temperature control” and “failure in pH control” were simulated by stopping the responsible control system of the respective bioreactors for approximately 1 h. We found a higher DCW at the end of the cultivation in C5; no differences were found to the reproducibility runs in the USP and DSP. The higher DCW resulted from a higher starting DCW at the start of the induction phase, because the non-induced fed-batch ran approximately 30 min longer (see Fig. S2, Fig. S3, and Fig. S4 for process and physiology data of cultivation C5, C6, and C7). In the USP, the loss of temperature control in C6 was followed by an immediate temperature increase from 30 to 40.4 °C at a rate of 0.17 °C min^−1^. Together with the temperature increase, the dO_2_ decreased and the offgas CO_2_ increased, respectively. During the deviation phase and at the end of the regeneration phase (= end of cultivation), no metabolites or glucose accumulated (Fig. 1). However, clear foam formation was visible during the deviation phase, which stopped when the temperature control was activated again. The physiological parameters did not show an irreversible change in cellular performance compared to the reference. Although foam formation might result from cell lysis, no additional indications, like a decreased DCW or an elevated absorption in 260 nm, were found (Table 4). It was rather believed that foam formation resulted from denatured extracellular protein and a decreased gas solubility (Henry’s law) through the increased temperature. The specific product titer was not affected, but, more interestingly, it had a rather positive impact on the following DSP (Fig. 2). We found less target protein in the IB wash solution than in the reference and the refolding yield of 47 ± 5% was clearly elevated.

**Table 4:** Results for the cultivations C5–C7

	C5 (reference)	C6 (*T* failure)	C7 (pH failure)
**Regeneration phase**			
Deviation phase [h]	0	1.0	1.0
DCW_End_ [g L^−1^]	49.0 ± 0.2	48.9 ± 0.2	50.7 ± 0.4
Glucose [g L^−1^]	0.20 ± 0.00	0.25 ± 0.00	1.08 ± 0.00
Acetate [g L^−1^]	0.61 ± 0.00	0.68 ± 0.00	0.32 ± 0.00
Formate [g L^−1^]	0.88 ± 0.00	0.87 ± 0.00	0.67 ± 0.00
Ethanol [g L^−1^]	0.15 ± 0.00	0.18 ± 0.00	0.12 ± 0.00
A260 [AU]	20.8 ± 0.1	20.8 ± 0.4	22.8 ± 0.0
*q*_s_ [g g^−1^ h^−1^]	0.22 ± 0.01	0.21 ± 0.01	0.21 ± 0.00
*Y*_X/S_ [Cmol Cmol^−1^]	0.38 ± 0.07	0.34 ± 0.08	0.25 ± 010
$Y_{{{\text{CO}}_{ 2} /{\text{S}}}}$ [Cmol Cmol^−1^]	0.51 ± 0.01	0.53 ± 0.01	0.53 ± 0.01
C-balance [Cmol Cmol^−1^]	0.92 ± 0.07	0.92 ± 0.08	0.91 ± 0.11
Specific product titer [mg g^−1^ WCW]	55.7 ± 3.5	60.3 ± 9.6	52.6 ± 3.5
**IB wash**			
Ratio target/total [–]	0.12 ± 0.01	0.09 ± 0.00	0.11 ± 0.01
Ratio IB/BM [–]	0.25 ± 0.01	0.25 ± 0.01	0.25 ± 0.01
**IB solubilisation**			
Duration [h]	1	1	1
Ratio target/total [–]	0.58 ± 0.01	0.58 ± 0.02	0.54 ± 0.06
**IB refolding**			
Duration [h]	3	3	3
Refolding [%]	34 ± 5	47 ± 5	29 ± 2
Ratio target/total [–]	0.18 ± 0.01	0.19 ± 0.01	0.19 ± 0.01

Results are given for the regeneration phase in the upstream process, which followed the deviation phase. Dry cell weight measurement errors were derived from triplicate measurements. Standard deviations from physiological parameters were derived from error propagation. Standard deviations from the specific product titer and the downstream process parameters were derived from duplicate processing and measuring. Shown downstream process focused on the inclusion body washing procedures (IB wash), the inclusion body solubilisation procedure (IB solubilisation), and the final refolding process (IB refolding)

In cultivation C7, the loss of pH control led to a decrease from pH 7.2 to 6.7 of a rate of 0.008 min^−1^. Again, no accumulation of metabolites or glucose was seen during the deviation phase. However, we observed an elevated absorption in 260 nm and an increased glucose content of 1 g L^−1^ in the culture broth at the end of the regeneration phase (= end of cultivation) (Fig. 1). Although the increased glucose content was probably related to a decreased cellular performance, no decreased DCW or decreased physiological parameters were found. The specific product titer and the DSP results showed no difference to the reference (Fig. 2). Therefore, the short inability to control the temperature or the pH seemed to have no negative impact on the USP and the DSP of the IB process.

## Introduction of technical failures at the end of induction phase

**Fig. 3:**
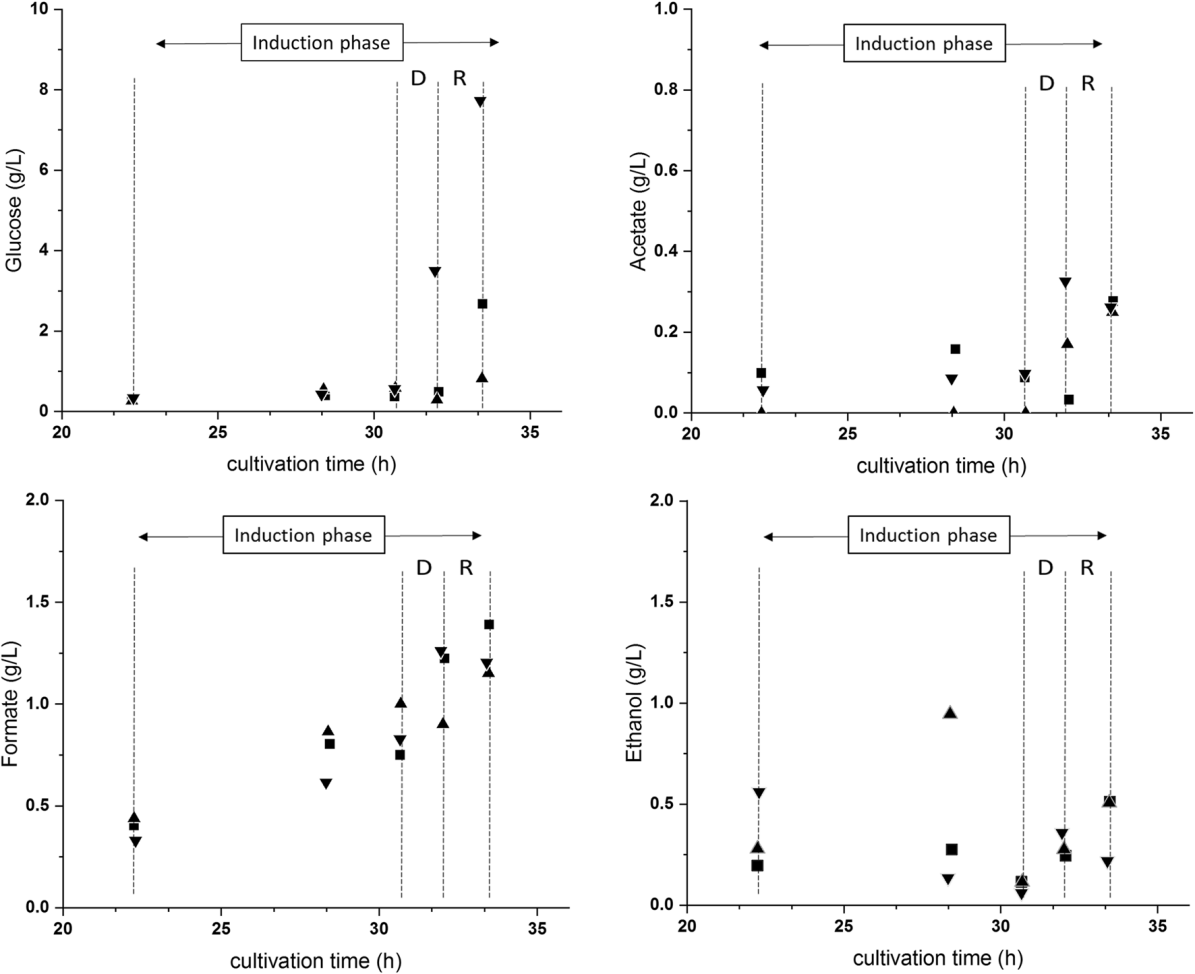
Monitoring of glucose and metabolite (ethanol, formate, and acetate) content in the cultivation broths of C8—C10. (Filled square) C8—reference run; (filled triangle) C9—interruption of feeding; (filled inverted triangle) C10—overfeeding. Induction phase is shown. Deviation phase is shown as D and regeneration phase as R. The induction phase included the phases D and R

**Fig. 4:**
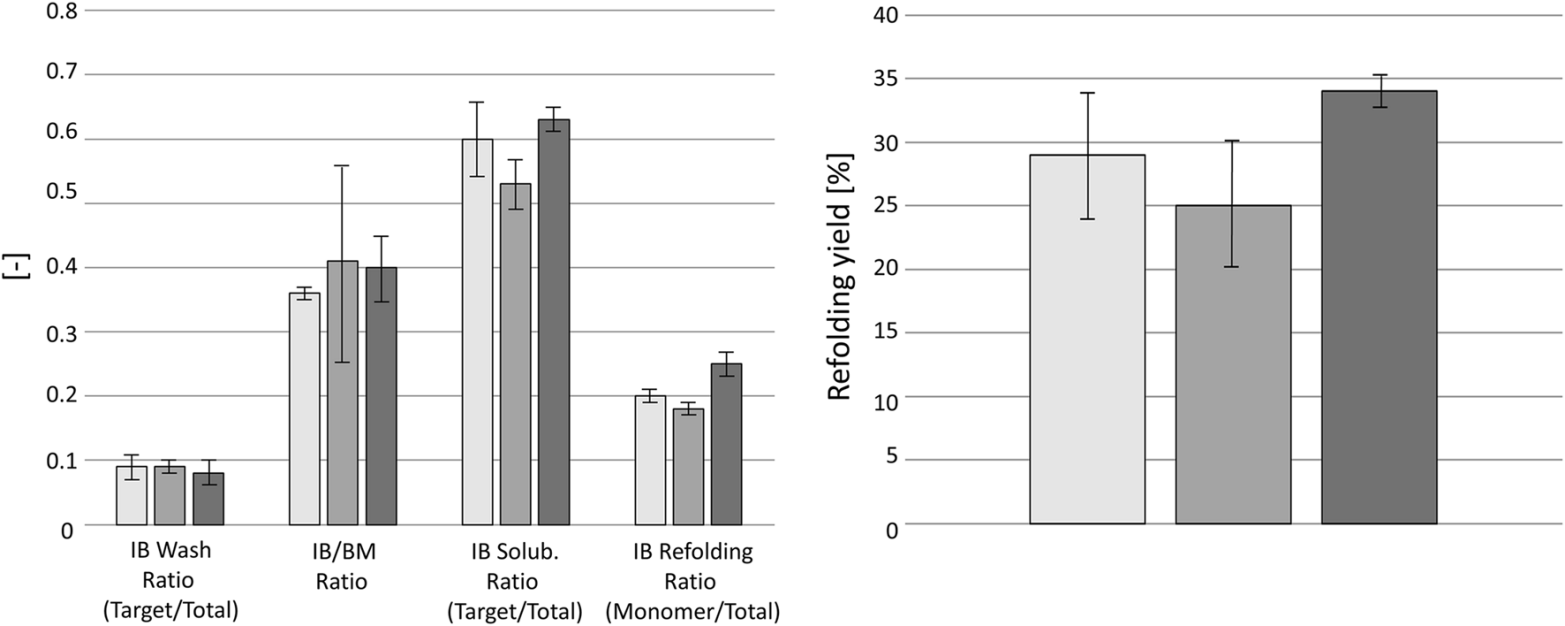
Evaluation of inclusion body downstream processing from cultivations C8—C10. Bars from left to right show results from C8—reference run, C9—interruption of feeding, and C10—overfeeding. On the left, the results of the inclusion body wash procedure, the measured ratio of inclusion body to biomass after the inclusion body wash, the purity of inclusion body solubilisation, and the purity of the inclusion body refolding procedure are shown. On the right, the final refolding yield after 180 min of refolding is shown. Error bars were derived from duplicates for all shown results

In the final stage of the induction phase, substrate depletion, due to an empty feed tank or overfeeding, due to the strains decreasing ability for substrate uptake, can occur. In cultivations C9 and C10, the technical failures “interruption of feeding” and “overfeeding” were simulated by stopping the responsible pump or increasing the feed addition for the respective bioreactors for approximately 1 h (see Fig. S5, Fig. S6, and Fig. S7 for process and physiology data of cultivation C8, C9, and C10). Subsequent to the interruption of feeding in C9, the dO_2_ indicated the immediate depletion of glucose. The cellular activity decreased and the pH increased as it was expected upon substrate depletion, because organic acids were most likely taken up. The pH increase was maintained by acid addition in the deviation phase. After standard process conditions were again present, the process parameters stabilized quickly. However, we found a decreased *Y*_X/S_, $Y_{{{\text{CO}}_{ 2} /{\text{S}}}}$ and C-balance during the regeneration phase compared to the reference (Table 5), but no accumulation of glucose or metabolites (Fig. 3). Together, these responses might highlight a metabolic switch towards substrate storage. In addition, neither the specific product titer nor the IB DSP was affected (Fig. 4).

**Table 5:** Results for the cultivations C8–C10

	C8 (reference)	C9 (feed stop)	C10 (overfed)
**Regeneration phase**			
Deviation phase [h]	0	1.4	1.4
DCW_End_ [g L^−1^]	44.7 ± 0.2	44.3 ± 0.3	45.2 ± 0.1
Glucose [g L^−1^]	2.68 ± 0.01	0.82 ± 0.00	7.73 ± 0.03
Acetate [g L^−1^]	0.28 ± 0.00	0.25 ± 0.00	0.26 ± 0.00
Formate [g L^−1^]	1.32 ± 0.00	1.15 ± 0.00	1.21 ± 0.00
Ethanol [g L^−1^]	0.51 ± 0.02	0.51 ± 0.00	0.22 ± 0.00
A260 [AU]	26.4 ± 0.1	26.6 ± 0.4	25.4 ± 0.0
*q*_s_ [g g^−1^ h^−1^]	0.19 ± 0.00	0.21 ± 0.00	0.17 ± 0.00
*Y*_X/S_ [Cmol Cmol^−1^]	0.26 ± 0.07	0.15 ± 0.03	0.26 ± 0.05
$Y_{{{\text{CO}}_{ 2} /{\text{S}}}}$ [Cmol Cmol^−1^]	0.59 ± 0.00	0.47 ± 0.00	0.64 ± 0.01
C-balance [Cmol Cmol^−1^]	0.94 ± 0.07	0.63 ± 0.04	0.95 ± 0.05
Specific product titer [mg g^−1^WCW]	81.2 ± 9.1	83.7 ± 3.7	88.9 ± 5.9
**IB wash**			
Ratio target/total [–]	0.09 ± 0.02	0.09 ± 0.01	0.08 ± 0.02
Ratio IB/BM [–]	0.36 ± 0.01	0.41 ± 0.17	0.40 ± 0.05
**IB solubilisation**			
Duration [h]	1	1	1
Ratio target/total [–]	0.60 ± 0.06	0.53 ± 0.04	0.63 ± 0.02
**IB refolding**			
Duration [h]	3	3	3
Refolding [%]	29 ± 5	25 ± 5	34 ± 1
Ratio target/total [–]	0.20 ± 0.01	0.18 ± 0.01	0.25 ± 0.02

Results are given for the regeneration phase in the upstream process, which followed the deviation phase. Dry cell weight measurement errors were derived from triplicate measurements. Standard deviations from physiological parameters were derived from error propagation. Standard deviations from the specific product titer and the downstream process parameters were derived from duplicate processing and measuring. Shown downstream process focused on the inclusion body washing procedures (IB wash), the inclusion body solubilisation procedure (IB solubilisation), and the final refolding process (IB refolding)

The increase in feeding in cultivation C10 led to unexpected results. Although additional available substrate should be visible in increased cellular activity, the expected increase in offgas CO_2_ and decrease in dO_2_ was not found in the deviation phase. In addition, the physiological parameters were not affected during the deviation phase. Furthermore, the increase in feeding led to accumulation of glucose (Fig. 3). Furthermore, glucose accumulation proceeded during the regeneration phase (Table 5). It rather seemed that the cellular machinery was not able to cope with the additional substrate and was already running at its maximum specific substrate uptake rate (*q*_s, Glc, max_). Acetate accumulation, which is a clear indicator of overflow metabolism, was also not present during the deviation and regeneration phase. The increased feed addition led to no increase in the specific product titer or an impact on the IB DSP (Fig. 4). Therefore, feeding-related technical failures at the end of the induction phase did not seem to have a negative impact on the USP and DSP.

## Discussion

The controlled introduction of technical failures revealed interesting aspects of the IB production chain in the USP and DSP. However, we have to highlight that we focused only on quantitative measurements like titer and purity and did not analyse the biological activity and CQAs of the antibody fragment. The initial evaluation of the whole process chain (C1–C4) was a valuable asset to determine the variability of each phase and unit operation. Almost all analysed parameters in the USP and DSP showed a $\varTheta$ < 10%, and hence, we concluded that the presented process was reproducible. However, we did observe increased standard deviations for a variety of parameters, like the *Y*_X/S_ and in the steps including IB processing, which was probably caused by human interaction. This problem is known especially for the USP and is tackled by researchers through development for sampling automation and closed-loop process control (e.g., [37, 38]). In this study, IB processing was only possible through human interaction, due to the given sample sizes. However, the goal of this study was not process optimization, but generation of process knowledge. Nevertheless, the found IB content of around 30% in the biomass stood in good agreement with recent findings in our research group. We have shown that the maximum intracellular IB size varied between 500 and 700 nm [16], which resembles a ratio around 30% of IB per cell given the rough *E. coli* size estimation of 2 µm. Furthermore, the basic and cheap refolding with glycerol as single additive in deionized water resulted in a refolding yield of around 30% compared to ~ 49% [39] or 32.3% [35] for similar proteins in more complex buffers.

### Impact of technical failures on the USP

Technical failures are critical in the phase of recombinant protein production, because they might change the cellular physiology and productivity. The four technical failures that we introduced in the induction phase resulted in varying responses. First, the loss in temperature control in cultivation C6 clearly increased the metabolic activity of the cells, which most likely also increased their *q*_s, Glc max_ for a short time. However, given that a regeneration phase under normal conditions was added, no lasting negative impact on cellular physiology or product quantity was found. Recently, constant induction temperatures of ~ 40 °C were reported to reduce the IB titer and target protein activity [15, 28]. The short shift in temperature had no negative impact on specific product titer, although it might be reasonable that longer shifts and higher temperatures lead to cell lysis and decrease in titer [15]. The loss of pH control in cultivation C7 neither led to foam formation nor clear changes in the dO_2_ or offgas values, but ongoing feed addition led to fast acidification of the culture broth to pH 6.7 in approximately 1 h. Again, no negative impact on the USP was found, including also the specific product titer compared to the reference, furthermore, recent results from our group highlighted a positive impact of pH < 7.0 on IB titer [15]. Although the pH decrease was only present for 1 h in our study, longer durations of pH decrease, especially below growth inhibiting conditions ~ pH 4.5 [40], would certainly have negative effects on the USP. In the next cultivation series (C8–C10), we focused on prolonged induction times (> 8 to 10 h) that are usually necessary to increase product yield. However, it is known that *E. coli* suffers from performance decreases, like decreased *µ* or *q*_s, Glc_, due to the metabolic stress upon recombinant protein production [32]. Therefore, we chose feeding-related technical failures to analyse substrate accumulating conditions, but also substrate depleting conditions. Substrate depletion could result from wrong calculations for feed volume, defect pumps, or tubes for feed addition. In our study, the substrate depletion led to no accumulation of stressor metabolites (acetate and formate). Interestingly, the observed decrease in physiological parameters (*Y*_X/S_, $Y_{{{\text{CO}}_{ 2} /{\text{S}}}}$, C-balance) and cell growth in the subsequent regeneration phase might be explained by recent findings regarding glycogen storage and consumption in *E. coli* [21]. It was reported that, upon depletion of substrate, *E. coli* cells switched their metabolism towards glycogen and acetate consumption for maintenance and vice versa, when substrate was available again. This would represent a rerouting of anabolism and catabolism and decrease *Y*_X/S_ and $Y_{{{\text{CO}}_{ 2} /{\text{S}}}}$. However, no negative impact on specific product titer was found. In contrary to C9, cultivation C10 experienced an increased feed addition in the deviation phase. There, glucose accumulation was increasing and dO_2_ and offgas values did not represent increased substrate metabolization. This highlighted that the cellular machinery was already running at its maximum capacity (*q*_s, Glc, max_) and was not able to metabolize additional substrate [32]. Similarly to cultivation C9, we could not observe a change in the specific product titer at the end of the cultivation, when compared to the reference. Summarizing, none of the presented technical failures should lead to process termination and batch loss, especially because no indications of cell lysis or decrease in specific product titer were found.

### Impact of technical failures on the DSP

The integrated approach in this study to analyse not only the USP, but also the subsequent IB DSP resulted in some unforeseen results that might even improve future IB processes. First and most importantly, we could not show that the introduced technical failures had a negative impact on the DSP. Neither the IB purity nor the refolding step was negatively affected by the introduced technical failures in the USP. Therefore, we recommend using the IB containing biomass in each case. Furthermore, we later observed an increased refolding yield for IBs from cultivation C6, in which the temperature increased up to 40 °C for a short time. This finding was an interesting addition to recent results from our research group that showed a negative impact of increased temperatures on IB purity and titer [15]. The short increase in temperature in C7 did not negatively affect specific product titer or purity in our study, but we hypothesized that the short temperature increase led to an increased content of partially folded protein in the IBs, which aided the final refolding process.

## Conclusion

Here, we presented a reproducible IB production process chain for an antibody fragment, which yielded high IB content, high specific product titer, and a good refolding yield of 30% under simplest conditions. The introduction of technical failures proved that the IB production process chain shows great robustness in the DSP, which is most probably derived from the IB properties that protect the target protein from intra- and extracellular influences. From our results, we can conclude that the controlled introduction of technical failures is an easy method to validate theoretical considerations from risk analysis and that it provides the possibility to find process-boosting parameter shifts that would have been neglected. In our case, the short increase in temperature clearly increased the refolding yield. Most importantly, we could show that the occurrence of such technical failures does not necessarily affect the USP and DSP negatively. Therefore, one does not have to discard the cultivation broth, but rather proceed with the IB DSP. We hope that our study provides reference data for researchers in academia and industry that work with bacterial IBs. This study marks the beginning of a series of similar studies, which we will perform with soluble recombinant proteins in *E. coli* and more complex organisms in the future.

## Electronic supplementary material

Below is the link to the electronic supplementary material.

Supplementary material 1 (DOCX 2605 kb)
