# Supplementary material for: The impact of technical failures during cultivation of an inclusion body process
Source: Bioprocess Biosyst Eng. 2019 Jul 2;42(10):1611–24. doi: 10.1007/s00449-019-02158-x (PMC6751153; doi:10.1007/s00449-019-02158-x)
Supplement: Supplementary file 1 — Supplementary material 1 (DOCX 2605 kb) [file 449_2019_2158_MOESM1_ESM.docx]

**Supplementary Materials**

**The impact of technical failures during cultivation of an inclusion body process**

Alexander Pekarsky^1^, Vanessa Konopek^1^ and Oliver Spadiut^1*^

^1^ Technische Universität Wien, Institute of Chemical, Environmental and Bioscience Engineering, Research Area Biochemical Engineering, Gumpendorfer Strasse 1a, 1060 Vienna, Austria

^*^ Correspondence: Oliver Spadiut, TU Wien, Institute of Chemical, Environmental and Bioscience Engineering, Research Area Biochemical Engineering, Gumpendorfer Strasse 1a, 1060 Vienna, Austria. Tel: +43 1 58801 166473, Fax: +43 1 58801 166980, Email: oliver.spadiut@tuwien.ac.at


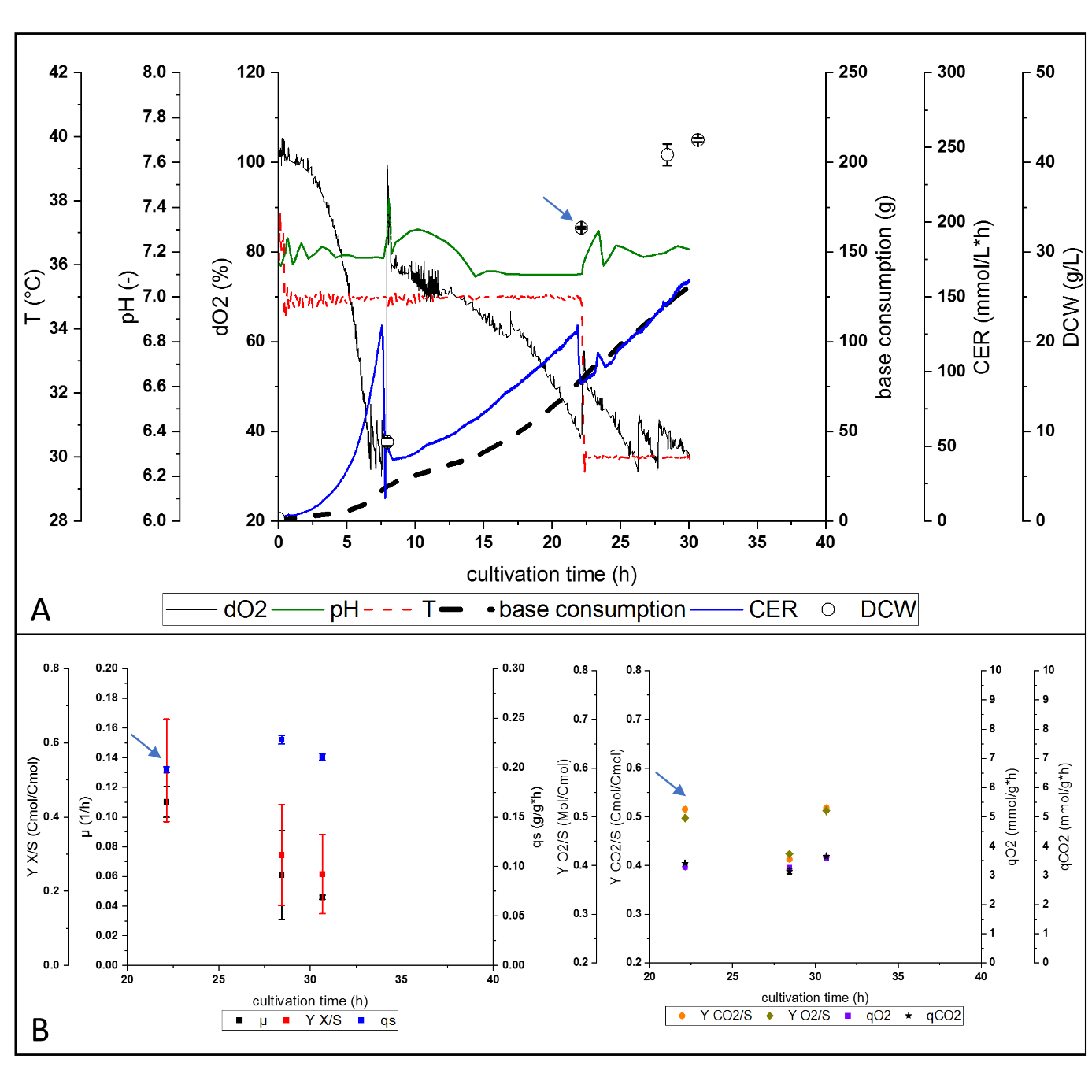


**Fig. S 1** Process and physiology data for cultivation C1. Start of induced fed-batch is marked by blue arrow. A) Process data of temperature (T), pH, dissolved oxygen (dO_2_), base consumption, carbon dioxide evolution rate (CER) and dry cell weight (DCW) is shown for batch, uninduced fed-batch and IPTG induced fed-batch phase. B) Physiology data of cultivation C1 for the uninduced fed-batch and the induced fed-batch: left: calculated data of biomass yield (Y_X/S_), specific growth rate (µ) and specific substrate uptake rate (q_s_) are shown; right: calculated data of oxygen consumption yield (Y_O2/S_), carbon dioxide evolution yield (Y_CO2/S_), specific oxygen uptake (q_O2_) and specific carbon dioxide evolution (q_CO2_) are shown.


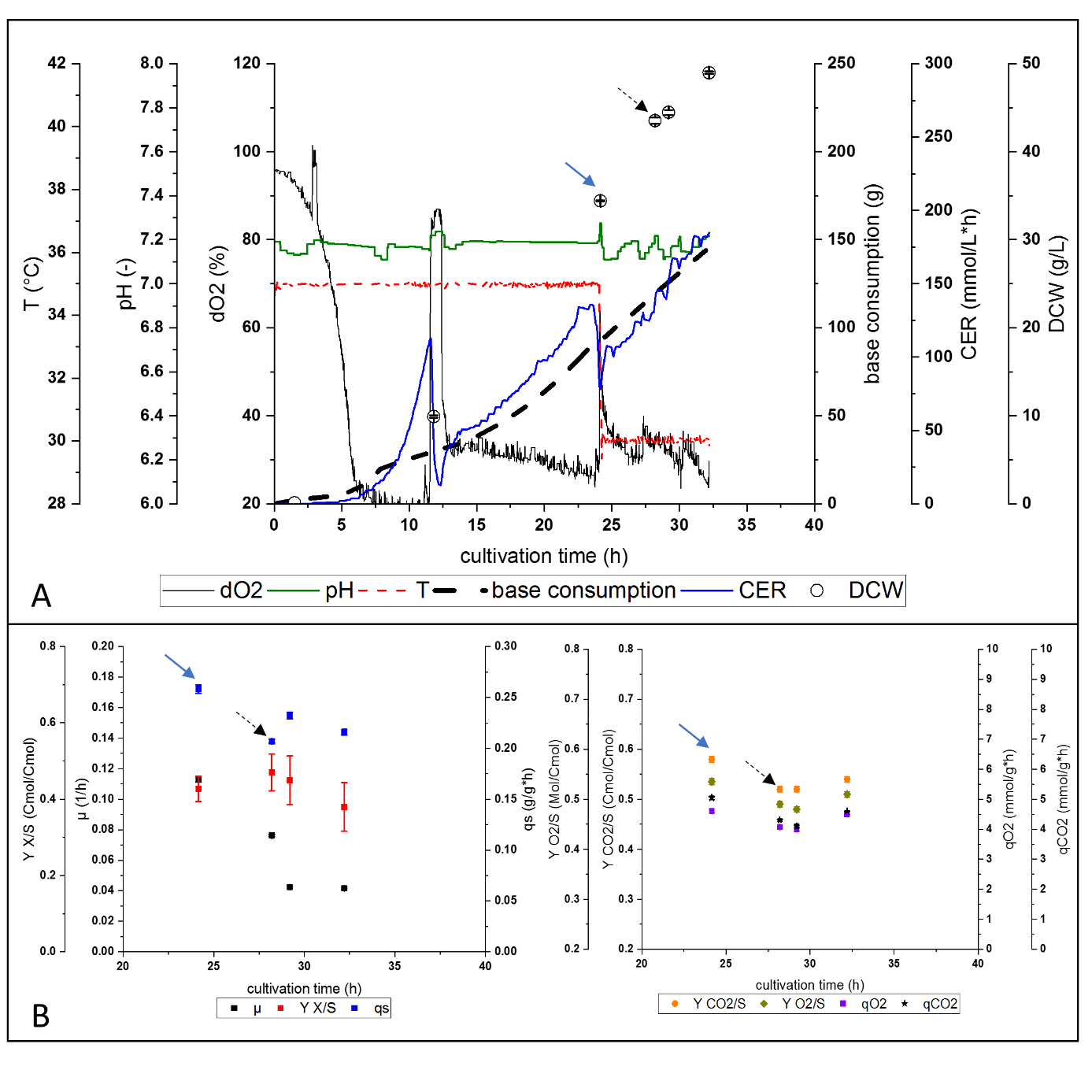


**Fig. S 2** Process and physiology data for cultivation C5 (reference). Start of induced fed-batch is marked by blue arrow, start of deviation through technical failure is marked by dashed black arrow. Herein, no technical failure was introduced. A) Process data of temperature (T), pH, dissolved oxygen (dO_2_), base consumption, carbon dioxide evolution rate (CER) and dry cell weight (DCW) is shown for batch, uninduced fed-batch and IPTG induced fed-batch phase. B) Physiology data of cultivation C5 for the uninduced fed-batch and the induced fed-batch: left: calculated data of biomass yield (Y_X/S_), specific growth rate (µ) and specific substrate uptake rate (q_s_) are shown; right: calculated data of oxygen consumption yield (Y_O2/S_), carbon dioxide evolution yield (Y_CO2/S_), specific oxygen uptake (q_O2_) and specific carbon dioxide evolution (q_CO2_) are shown.


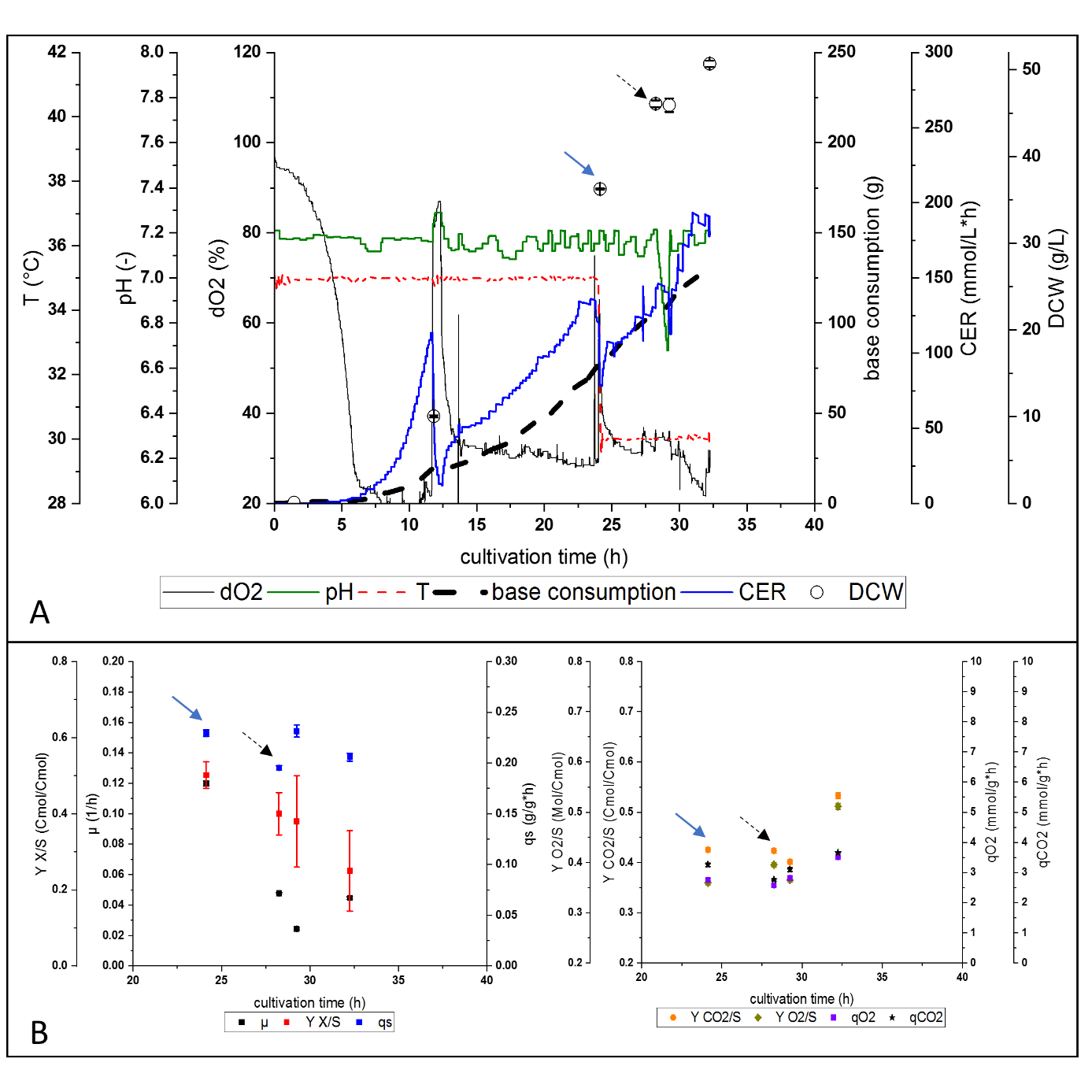


**Fig. S 3** Process and physiology data for cultivation C6 (T failure). Start of induced fed-batch is marked by blue arrow, start of deviation through technical failure is marked by dashed black arrow. Herein, the temperature control stopped working for appr. 1 h, which was followed by a regeneration phase under standard conditions. A) Process data of temperature (T), pH, dissolved oxygen (dO_2_), base consumption, carbon dioxide evolution rate (CER) and dry cell weight (DCW) is shown for batch, uninduced fed-batch and IPTG induced fed-batch phase. B) Physiology data of cultivation C6 for the uninduced fed-batch and the induced fed-batch: left: calculated data of biomass yield (Y_X/S_), specific growth rate (µ) and specific substrate uptake rate (q_s_) are shown; right: calculated data of oxygen consumption yield (Y_O2/S_), carbon dioxide evolution yield (Y_CO2/S_), specific oxygen uptake (q_O2_) and specific carbon dioxide evolution (q_CO2_) are shown.


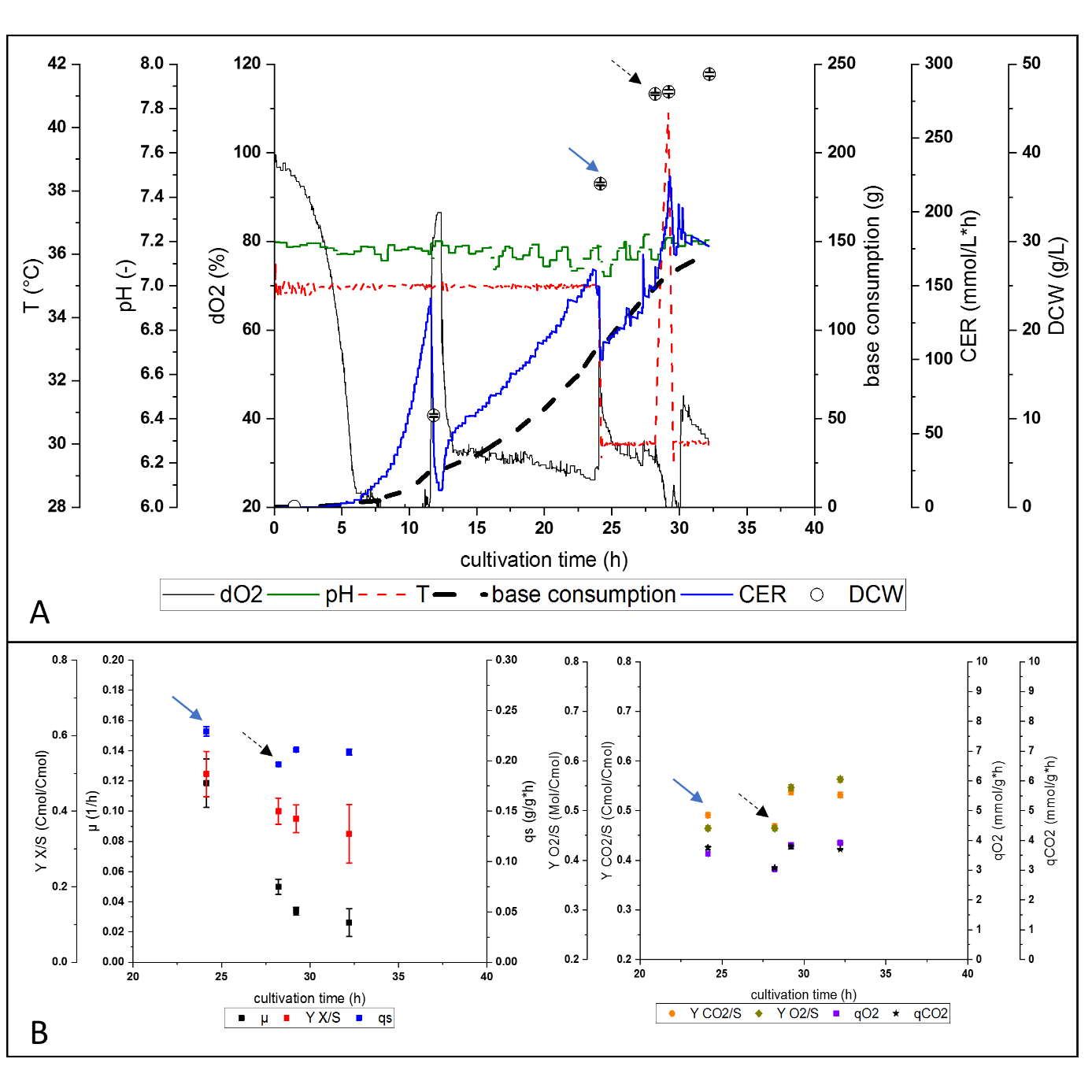


**Fig. S 4** Process and physiology data for cultivation C7 (pH failure). Start of induced fed-batch is marked by blue arrow, start of deviation through technical failure is marked by dashed black arrow. Herein, the pH control stopped working for appr. 1 h, which was followed by a regeneration phase under standard conditions. A) Process data of temperature (T), pH, dissolved oxygen (dO_2_), base consumption, carbon dioxide evolution rate (CER) and dry cell weight (DCW) is shown for batch, uninduced fed-batch and IPTG induced fed-batch phase. B) Physiology data of cultivation C7 for the uninduced fed-batch and the induced fed-batch: left: calculated data of biomass yield (Y_X/S_), specific growth rate (µ) and specific substrate uptake rate (q_s_) are shown; right: calculated data of oxygen consumption yield (Y_O2/S_), carbon dioxide evolution yield (Y_CO2/S_), specific oxygen uptake (q_O2_) and specific carbon dioxide evolution (q_CO2_) are shown.


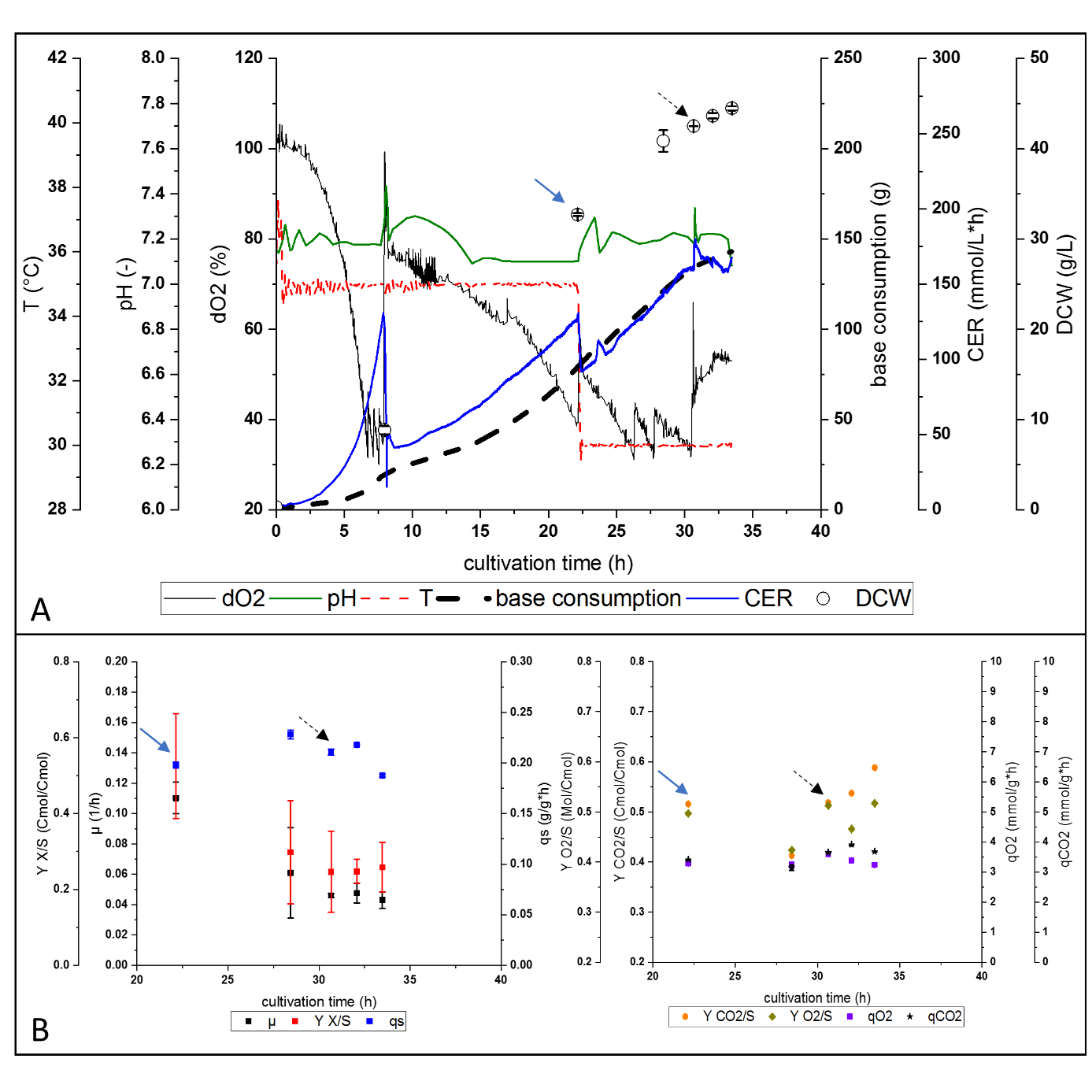


**Fig. S 5** Process and physiology data for cultivation C8 (reference). Start of induced fed-batch is marked by blue arrow, start of deviation through technical failure is marked by dashed black arrow. Herein, no technical failure was introduced. A) Process data of temperature (T), pH, dissolved oxygen (dO_2_), base consumption, carbon dioxide evolution rate (CER) and dry cell weight (DCW) is shown for batch, uninduced fed-batch and IPTG induced fed-batch phase. B) Physiology data of cultivation C8 for the uninduced fed-batch and the induced fed-batch: left: calculated data of biomass yield (Y_X/S_), specific growth rate (µ) and specific substrate uptake rate (q_s_) are shown; right: calculated data of oxygen consumption yield (Y_O2/S_), carbon dioxide evolution yield (Y_CO2/S_), specific oxygen uptake (q_O2_) and specific carbon dioxide evolution (q_CO2_) are shown.


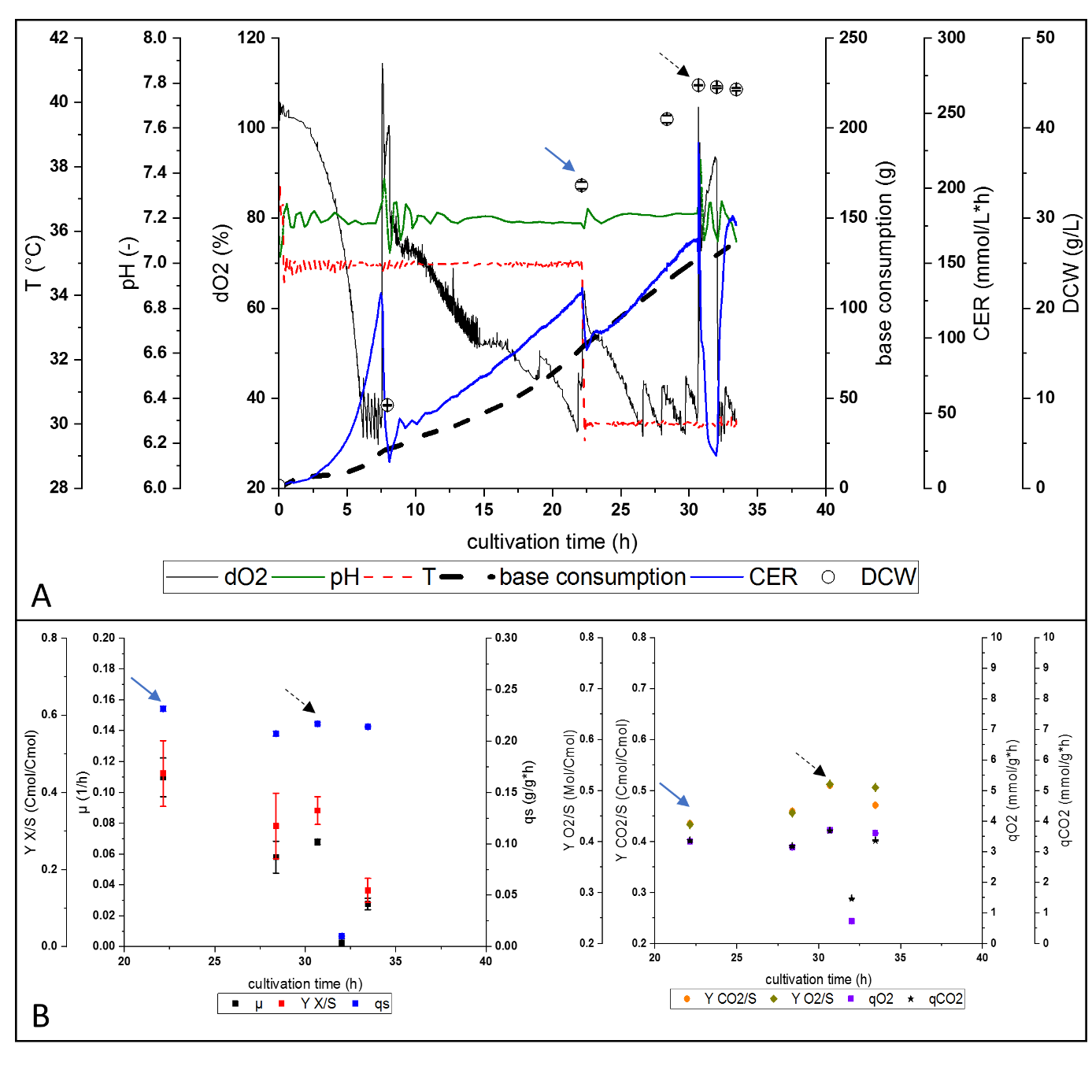


**Fig. S 6** Process and physiology data for cultivation C9 (feed stop). Start of induced fed-batch is marked by blue arrow, start of deviation through technical failure is marked by dashed black arrow. Herein, the feeding was stopped for appr. 1 h, which was followed by a regeneration phase under standard conditions. A) Process data of temperature (T), pH, dissolved oxygen (dO_2_), base consumption, carbon dioxide evolution rate (CER) and dry cell weight (DCW) is shown for batch, uninduced fed-batch and IPTG induced fed-batch phase. B) Physiology data of cultivation C9 for the uninduced fed-batch and the induced fed-batch: left: calculated data of biomass yield (Y_X/S_), specific growth rate (µ) and specific substrate uptake rate (q_s_) are shown; right: calculated data of oxygen consumption yield (Y_O2/S_), carbon dioxide evolution yield (Y_CO2/S_), specific oxygen uptake (q_O2_) and specific carbon dioxide evolution (q_CO2_) are shown. The yield coefficients for the sampling point (sampling point 4) after the deviation phase are not visible, because no substrate was added and therefore, no calculation was possible.


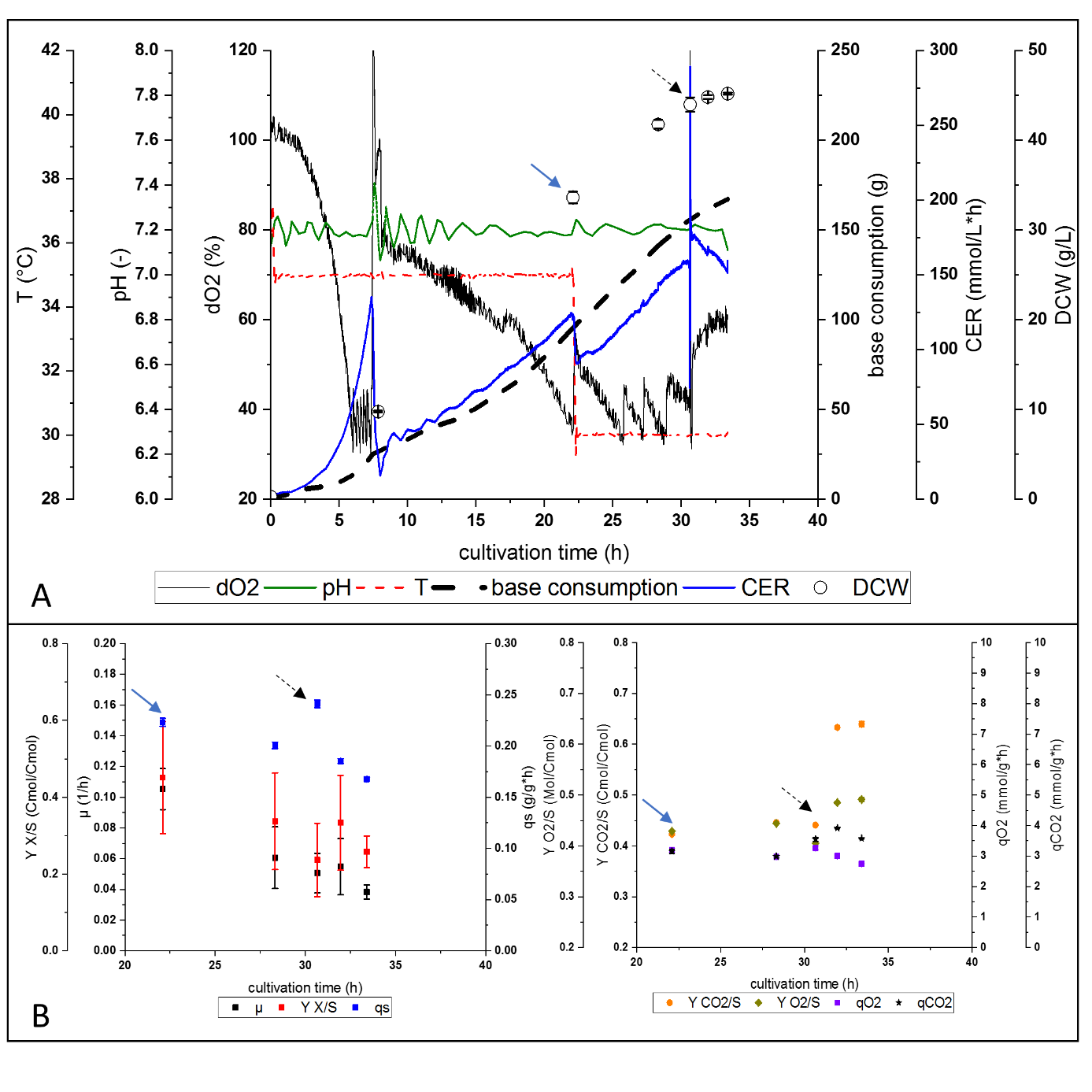


**Fig. S 7** Process and physiology data for cultivation C10 (overfeeding). Start of induced fed-batch is marked by blue arrow, start of deviation through technical failure is marked by dashed black arrow. Herein, the the cultivation was overfed for appr. 1 h, which was followed by a regeneration phase under standard conditions. A) Process data of temperature (T), pH, dissolved oxygen (dO_2_), base consumption, carbon dioxide evolution rate (CER) and dry cell weight (DCW) is shown for batch, uninduced fed-batch and IPTG induced fed-batch phase. B) Physiology data of cultivation C10 for the uninduced fed-batch and the induced fed-batch: left: calculated data of biomass yield (Y_X/S_), specific growth rate (µ) and specific substrate uptake rate (q_s_) are shown; right: calculated data of oxygen consumption yield (Y_O2/S_), carbon dioxide evolution yield (Y_CO2/S_), specific oxygen uptake (q_O2_) and specific carbon dioxide evolution (q_CO2_) are shown.
